# Barriers and Facilitators to Genetic Testing for Familial Hypercholesterolemia in the United States: A Review

**DOI:** 10.3390/jpm9030032

**Published:** 2019-07-01

**Authors:** Rachele M. Hendricks-Sturrup, Kathleen M. Mazor, Amy C. Sturm, Christine Y. Lu

**Affiliations:** 1 Department of Population Medicine, Harvard Pilgrim Health Care Institute and Harvard Medical School, Boston, MA 02215 USA; 2 Meyers Primary Care Institute, Worcester, MA 01605, USA; 3 Department of Medicine, University of Massachusetts Medical School, Worcester, MA 01655, USA; 4 Genomic Medicine Institute, Geisinger, Danville, PA 17822, USA

**Keywords:** familial hypercholesterolemia, genetic testing, implementation framework, barriers and facilitators, genomic medicine

## Abstract

Familial Hypercholesterolemia (FH) is an underdiagnosed condition in the United States (US) and globally, affecting an estimated 1/250 individuals. It is a genetic risk factor for premature cardiovascular disease and is responsible for an estimated 600,000 to 1.2 million preventable vascular events. Studies show that FH genetic testing can identify a causal gene variant in 60 to 80% of clinically suspected FH cases. However, FH genetic testing is currently underutilized in clinical settings in the US despite clinical recommendations and evidence supporting its use. Reasons for underutilization are not well understood. We conducted a literature review in the PubMed/MEDLINE database and eight peer-reviewed journals. After filtering for and reviewing 2340 articles against our inclusion criteria, we included nine commentaries or expert opinions and eight empirical studies reported between January 2014 and March 2019 in our review. After applying the Consolidated Framework for Implementation Research (CFIR), we identified a total of 26 potential barriers and 15 potential facilitators (estimated barrier to facilitator ratio of 1.73). We further estimated ratios of potential barriers to facilitators for each CFIR domain (Characteristics of Intervention, Outer Setting, Inner Setting, Characteristics of Individuals, and Process). Findings derived from our systematic approach to the literature and calculations of estimated baseline ratios of barriers and facilitators can guide future research to understand FH genetic testing implementation in diverse clinical settings. Our systematic approach to the CFIR could also be used as a model to understand or compare barriers and facilitators to other evidence-based genetic testing processes in health care settings in the US and abroad.

## 1. Introduction

Familial Hypercholesterolemia (FH) is an underdiagnosed condition in the United States (US) and abroad and a genetic risk factor for premature cardiovascular disease (CVD) [1,2,3,4,5]. CVD is currently the leading cause of preventable death for people of most ethnicities in the US [6]. FH is hallmarked by individual symptoms related to and signs of elevated low-density lipoprotein (LDL) cholesterol [1,2,3,4,5]. FH alone is responsible for an estimated 600,000 to 1.2 million preventable vascular events and affects an estimated 1/250 individuals in the US [1,2,3,4,5,7]. 

Studies show that FH genetic testing can identify a causal gene variant in 60 to 80% of clinically suspected FH cases and that large-scale DNA sequencing can identify FH cases that were either not clinically detected or potentially missed using an algorithmic approach [1,8]. A recent expert consensus panel recommended that FH genetic testing become the standard of care for patients with definite or probable FH [9,10]. Moreover, FH genetic testing is recommended in cases where results could change or influence patient management or when at-risk family members could benefit from testing and acknowledgement of their test results [10]. Studies show that testing can lead to improved FH diagnosis; initiated, continued, or improved adherence to treatment; improved LDL and total cholesterol levels; the provision of genetic counseling services; and patient education on lifestyle and daily management [8,11,12,13,14,15,16,17,18,19,20]. Also, the US Centers for Disease Control and Prevention (CDC) endorse FH genetic testing implementation because testing has the potential to positively and significantly impact public health [21,22]. The CDC currently provides a Genomics Application Toolkit to guide the implementation of FH genetic testing for those with clinical signs and family histories of CVD [23,24]. 

FH genetic testing, however, is underutilized in clinical practice in the US, with testing being used primarily in research (versus clinical) settings [9,20,25]. A recent survey among members of the American College of Cardiology showed that only 25% of primary care providers (PCPs), 24% of cardiologists, and 15% of cardiovascular team members use FH genetic testing to guide diagnosis [26]. Reasons for the observed FH genetic test underutilization are unclear; however, the fragmented and market-driven US health care system, public and private insurer willingness to pay, and a reported shortage of genetics specialists in rural US regions might contribute to the reported underutilization [27,28,29,30,31]. 

In a recent commentary, we (R.M.H.-S. and C.Y.L.) discussed seven key implementation challenges to FH genetic testing in the US [25]. In this review, we have applied the Consolidated Framework for Implementation Research (CFIR) to identify possible implementation barriers and facilitators to FH genetic testing in the US. Our findings are based on expert commentary, reviews, and empirical studies published in peer-reviewed literature. 

## 2. Methods

### 2.1. Search Strategy, Literature Selection, and Inclusion Criteria

In March 2019, articles were electronically searched in the PubMed/MEDLINE database. To control for potential PubMed/MEDLINE index discrepancies, identify articles that do not contain keywords (e.g., expert commentary), and overcome possible limitations to searching for relevant literature in a single public database, we also searched for articles directly in eight relevant peer-reviewed journals [32]. We selected eight specific peer-reviewed journals that have recently published articles related to the clinical management of familial hypercholesterolemia, based on the authors’ prior scoping of the literature. Five search strings were used to identify relevant expert commentary, case studies, literature reviews, and empirical studies. We limited our search to reports published between 2014 to 2019 to capture the latest, most timely, and relevant information. Our full search methodology and inclusion criteria are described in Table 1. The terms “urban” and “rural” were used in two search strings to capture literature that describes possible factors that might affect physical access to FH genetic testing. 

One author (R.M.H.-S.) performed the literature search, screened titles and abstracts, and reviewed full articles to determine topic relevance for information extraction. Literature searches were conducted separately using each search string in PubMed/MEDLINE and each of the eight selected journals. Duplicate articles were removed. After screening titles and abstracts, further articles were excluded due to topic irrelevance.

### 2.2. Choice of Implementation Framework

The CFIR is a flexible analysis framework that consists of five major domains (characteristics of intervention, outer setting, inner setting, characteristics of individuals, and process) and 39 constructs. CFIR domains are listed and defined in Table 2. These domains and constructs are being used to garner a current understanding of barriers and facilitators to the implementation of other evidence-based genomic technologies into standard medical practice [33,34,35,36]. We intend to use the CFIR to guide the leveraging of any identified facilitators and reduction or mitigation of any identified barriers to refine the FH genetic testing implementation process in diverse settings. 

The process for selecting implementation theories by scientists as of recent has been described as ‘haphazard’ and primarily driven by convenience or familiarity [37]. The CFIR is currently guiding the exploration of implementation barriers and facilitators to Universal Lynch Syndrome screening mechanisms that involve evidence-based and guideline-recommended genetic testing [33]. Thus, after considering this and closely reviewing several implementation frameworks, we determined that the CFIR would likely facilitate a careful identification and an organized evaluation of complex factors that could influence the implementation of FH genetic testing [37,38].

### 2.3. Information Extraction

One author (R.M.H.-S.) extracted information from relevant articles and categorized the information under a CFIR domain. When deemed appropriate, a single identified barrier or facilitator was categorized to more than one domain. Predefined CFIR domain definitions (see definitions in Table 2) guided the categorization. The extracted information and subsequent categorizations under each CFIR domain were reviewed by the second, third, and fourth authors (C.Y.L., K.M.M., and A.C.S.).

### 2.4. Analysis of Barriers and Facilitators

After reviewing each article that met the inclusion criteria, we identified and extracted relevant information that could be considered a potential barrier or facilitator. We coded each barrier and facilitator to an appropriate CFIR domain based on that CFIR domain’s definition. To gain a numerical estimate of barriers to facilitators, we calculated the ratio of potential barriers and facilitators identified for each CFIR domain and overall across all CFIR domains. 

## 3. Results

**Figure 1:**
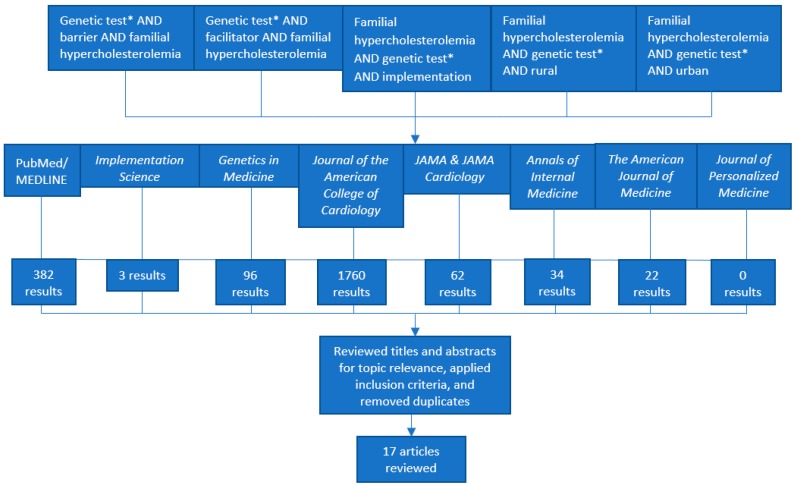
Literature Search Strategy and Results.

A total of 2359 results were returned (search strategy in Figure 1). After excluding 2340 articles due to irrelevance, applying our inclusion criteria, and removing duplicates, possible barriers and facilitators to FH genetic testing were extracted from a total of 17 articles. Twelve articles from our search in PubMed/MEDLINE, two articles from our search in Journal of the American College of Cardiology, and three articles from our search in Journal of the American Medical Association (JAMA) and JAMA Cardiology met our inclusion criteria. The articles identified and included in the review from American College of Cardiology, JAMA, and JAMA Cardiology were not found in PubMed/MEDLINE.

Potential barriers and facilitators were ultimately identified and extracted from 17 articles (nine commentaries or expert opinions and eight empirical studies; see Table 3). In total, 26 potential barriers and 15 potential facilitators to FH genetic testing in the US were identified. We thus estimate that the current overall ratio of potential barriers to facilitators to FH genetic testing in the US is 26/15 or 1.73.

### 3.1. CFIR Domain #1: Characteristics of Intervention

Under CFIR Domain #1, Characteristics of Intervention (FH genetic testing itself being the intervention of focus), many facilitators were related to meeting FH diagnosis criteria, DNA sample collection methods, insurance coverage, the availability of genetic counseling services, timeline for returning test results, and the interpretation and use of test results. Barriers under CFIR Domain #1 focused on the monetary costs of FH genetic testing, little access to testing services, concerns about privacy and discrimination, lack of insurance coverage for testing, and issues with recruiting or identifying family members for cascade testing. The estimated ratio of potential barriers to facilitators for CFIR #1 based on the literature is 9/11 or 0.82.

### 3.2. CFIR Domain #2: Outer Setting

Under CFIR Domain #2, Outer Setting, facilitators centered on physical access to testing services and expert consensus to support the use of FH genetic testing in cases of probable or definite FH. Barriers under CFIR Domain #2 were similar to those listed under CFIR Domain #1: recruitment of family members for testing, proximal access to services, concerns about privacy and discrimination, and coverage of costs for FH genetic testing. The estimated ratio of potential barriers to facilitators for CFIR #2 based on the literature is 6/3 or 2.00.

### 3.3. CFIR Domain #3: Inner Setting

Under CFIR Domain #3, Inner Setting, facilitators centered on the use of electronic health records (EHRs) to identify probands and relatives, and collaboration among clinicians. Barriers centered on lack of optimized diagnostic criteria for FH patient identification in EHRs, suboptimal time for assessing family history during clinic visits, less than optimal accuracy of family history reporting, and the poor adoption of paper-based and web-based tools that would help patients organize their family history outside of the clinical visit. The estimated ratio of potential barriers to facilitators for CFIR #3 based on the literature is 4/4 or 1.00.

### 3.4. CFIR Domain #4: Characteristics of Individuals

Under CFIR Domain #4, Characteristics of Individuals, facilitators were patient-focused, centering on the use of genetic counseling before and after FH genetic testing, patient readiness to undergo FH genetic testing, patient diagnosis of FH, and patient use of educational materials about FH. Barriers centered on patient concerns and knowledge about FH, low patient readiness to receive genetic test results, and providers perceiving FH genetic as outside of their scope of medical practice. The estimated ratio of potential barriers to facilitators for CFIR #4 based on the literature is 6/4 or 1.50.

### 3.5. CFIR Domain #5: Process

Under CFIR Domain #5, Process, facilitators centered on coordinated efforts among clinicians involved in the FH genetic testing process and the use of diagnosis codes to facilitate this process. The one barrier centered on the fact that FH risk stratification and subsequent clinical management is mostly guided by FH phenotypes (e.g., LDL cholesterol levels and therapeutic response) versus genotype. The estimated ratio of potential barriers to facilitators for CFIR #5 based on the literature is 1/4 or 0.25.

## 4. Discussion

To date, no single literature review has sought to identify possible barriers and facilitators to FH genetic testing using the CFIR framework. Moreover, no single analysis has involved the use of ratios to evaluate the presence of the potential barriers and facilitators to FH genetic testing in the US. Future empirical work should confirm the presence or absence of these potential barriers and facilitators across diverse health systems and settings.

Our decision to involve ratios of barriers and facilitators in our analysis was inspired by Gleacher et al., who assessed barriers and facilitators to the implementation of an electronic measurement feedback system and used ratios as a quantitative measure to compare the mean number of barriers or facilitators reported by clinicians at two clinics [51]. To control for the possibility that clinics with greater program implementation may report many more barriers than clinics with lower program implementation and to account for a possibly unequal number of clinicians across those sites, Gleacher et al. took the average (versus total) number of barriers and facilitators reported by each clinician interviewed from each clinic [51]. The necessity of this control measure is grounded in the fact that high implementing clinics are likely to encounter more challenges, given their relatively larger amount of experiences in and exposure to working through the challenges of implementing the new practice. Lyon and Lewis noted, however, an important limitation to this approach; some categories of contextual factors captured within each identified barrier and facilitator could vary in weight or in their individual ability to influence an implementation setting [52]. One possible mechanism to address this limitation in future research is by using configurational comparative methods (e.g., Qualitative Comparative Analysis and coincidence analysis) as analytical techniques to assess causal complexity in organizational-level process or resource implementation [33]. Future work involving the CFIR could examine barriers and facilitators to FH genetic testing using Gleacher et al.’s approach for these same reasons.

Also, since health care in the US is largely fragmented (versus integrated) and because patients can be denied insurance coverage of genetic testing (when testing is considered investigational versus medically necessary), the number of barriers and facilitators experienced by clinicians could significantly vary based on factors that include but are not limited to geographic location; provider knowledge, attitudes, and beliefs about genetic testing; and insurance payor coverage policies. The estimated overall ratio of barriers to facilitators we have attained of 1.73 can be used as a numerical benchmark for future studies that might measure improvement (i.e., achieving a higher average barrier/facilitator) or as a benchmark or baseline measure that can be used by health systems seeking to implement FH genetic testing. For example, a health system with several facilitators on our list, but only a few of the barriers on our list, will have an overall average barrier/facilitator ratio that is lower than our baseline ratio of 1.73.

Researchers are currently using the CFIR as a guide to explore barriers and facilitators to genetic testing for other conditions with Tier 1 genomic applications according to the CDC (e.g., Lynch syndrome) [24,33]. The Implementing GeNomics In PracTicE Network Common Measures Working Group (IGNITE-CMG), established by the National Human Genome Research Institute in 2013, is using the CFIR to systematically identify constructs and measures that are relevant to the holistic evaluation of genomic medicine, standardization of data collected across clinical genomics projects, and combining of relevant data into a centralized resource for analyses across multiple networks [34]. Thus, our decision to apply the CFIR to identify and understand barriers and facilitators to FH genetic testing is consistent with the greater research community.

Keith et al. applied the CFIR and conducted practice interviews and visits to assess implementation barriers and facilitators to clinic participation in a program consisting of five functional components. By cross-referencing, in a two-way matrix, the five program components to each CFIR domain, they were able to understand the influence of program implementation barriers and facilitators among 21 small, medium, and large practices [53]. The FH genetic testing process also involves five functional areas or components: (1) identifying an FH index patient, (2) providing or referring patients to genetic counseling services, (3) offering genetic testing services, (4) educating patients about genetic test results, and (5) engagement in recommended clinical screening and care [9]. Thus, using Keith et al.’s analysis approach, the CFIR can be used to delineate and understand the influence of various barriers and facilitators found across the five components to the FH genetic testing process [9].

One key limitation to this review could be our use of the CFIR as a guiding framework for our analysis. The IGNITE-CMG described two key limitations to using the CFIR as a guiding framework to understand the implementation of genomic technologies: (1) the CFIR does not fully capture all domains pertinent to genomic medicine and (2) the CFIR is specifically focused on factors relevant to the health system rather than local community values [34]. They addressed these limitations by expanding the core CFIR structure to incorporate domains that are relevant to patients, families, and local communities. They explained that, in genomic medicine, patient values and local culture are considerably important and underscored by efforts dedicated to understanding patient perceptions, anxiety, and personal utility, especially in cases where patients who are generally healthy [34]. The cost of FH genetic testing, followed by ethical, legal, and social concerns about FH genetic testing (e.g., stigmatization or discrimination by health insurers, privacy over genetic information, etc.) were prevalent barriers identified in our analysis. Also, FH patient-centric educational tools and decision aids along with FH patient readiness to engage in behavioral changes needed for treatment were among the identified facilitators. These barriers and facilitators could relate to patient values and local culture. Thus, a closer analysis of such barriers and facilitators within and across diverse settings and populations, where patient values and local culture might vary, could provide clues as to whether CFIR structure expansion is needed. Despite this key limitation of using the CFIR, we have described how the CFIR might be used to signal the presence of possible barriers that could be rooted in patient values and local culture.

Other limitations to this review include limiting the search strategy to only a single database and eight peer-reviewed journals; it possible that other potentially relevant publications in unsearched peer-reviewed journals or databases might have been missed (e.g., Journal of Genetic Counseling). Further, our list of barriers/facilitators is not based on empirical studies alone, as we also included expert commentary to generate a relatively comprehensive list of potential barriers and facilitators to FH genetic testing. Expert commentaries do not often include searchable keywords. Thus, it is possible that commentary articles included in our review from the selected journals were not identifiable in PubMed/MEDLINE using keywords from our search strategy.

FH genetic testing has been reported within recent years in diverse locations and health systems within and outside of the US and within adult and pediatric populations (e.g., Australia, India, China, and countries in Europe) [11,12,13,14,15,16,17,18,19,20,54]. Future work might also involve the CFIR to describe or compare potential barriers and facilitators to FH genetic testing in countries or locations outside of the US. Future work could also compare barriers and facilitators to FH genetic testing in socialized versus non-socialized health care systems and settings.

FH, Lynch syndrome, and breast cancer are diseases considered by the CDC as having sufficient evidence to support the implementation of genetic testing for clinical diagnosis [24]. As mentioned, researchers are currently using the CFIR to identify implementation barriers and facilitators to Universal Lynch Syndrome screening [33]. However, there is a paucity of literature describing how the CFIR can be applied to understand the scope of current barriers and facilitators to evidence-based genetic testing for breast cancer-related genes (BRCA 1/2) [33]. Variations in payor coverage in the US for multi-panel genetic testing for cancer-related genes render it likely that cost- or access-related factors could serve as implementation barriers or facilitators to cancer-related genetic testing [30]. There is, thus, opportunity for future research to apply the CFIR to examine such barriers or facilitators to evidence-based genetic testing for breast or other cancer-related genes. Future work might also assess implementation barriers and facilitators to evidence-based practices within and outside of medical genetics.

## 5. Conclusions

Our work demonstrates how the CFIR can guide a systematic analysis of the literature to identify potential barriers and facilitators to FH genetic testing in the US. Our systematic approach might also guide the exploration or comparison of barriers and facilitators to other evidence-based genetic testing programs, such as genetic testing for breast cancer-related genes, in diverse health systems and geographic locations outside of the US. We identified a total of 26 potential barriers and 15 potential facilitators to FH genetic testing to calculate an estimated barrier to facilitator ratio of 1.73. Strategies should be taken to reduce the number of barriers and leverage current facilitators to FH genetic testing in the US to increase the likelihood of observing the clinical benefits associated with FH genetic testing. Moreover, future research should consider our findings as a crucial starting point to assess how these potential barriers and facilitators impact key steps within the FH genetic testing process.

## Figures and Tables

**Table 1:** Search Strategy: Keywords, Search Strings, Databases and Journals, and Inclusion Criteria.

Keywords/Strings Used for Literature Search	Databases and Journals	Inclusion Criteria
genetic test* AND barrier AND familial hypercholesterolemia genetic test* AND facilitator AND familial hypercholesterolemia familial hypercholesterolemia AND genetic test* AND implementation familial hypercholesterolemia AND genetic test* AND rural familial hypercholesterolemia AND genetic test* AND urban	PubMed/MEDLINE Implementation Science Genetics in Medicine Journal of the American College of Cardiology Journal of the American Medical Association (JAMA) JAMA Cardiology Annals of Internal Medicine The American Journal of Medicine Journal of Personalized Medicine	English language Written or reported by investigators based in the United States Human studies Empirical studies Case studies, expert commentaries, and literature reviews Published within the last 5 years (2014–2019)

**Table 2:** Consolidated Framework for Implementation Research (CFIR) Domain Definitions.

CFIR Domain	
1. Characteristics of Intervention	Key attributes of interventions influence the success of implementation. Includes adaptability, complexity, cost, design quality and packaging, evidence strength and quality, intervention source, relative advantage, and trialability.
2. Outer Setting	External influences including cosmopolitanism, external policies and incentives, patient needs and resources, and peer pressure.
3. Inner Setting	Active interacting facets within a setting that include structural characteristics, networks and communications, culture, implementation climate, and readiness for implementation.
4. Characteristics of Individuals	The actions and behaviors of individuals. Includes individual identification with an organization, individual stage of change, knowledge and beliefs about the intervention, other personal attributes, and self-efficacy.
5. Process	The process of implementing an intervention. Includes or involves behaviors of engagement, execution, planning, reflecting, and evaluation.

Note: CFIR domain definitions taken from https://cfirguide.org.

**Table 3:** Possible Barriers and Facilitators to Genetic Testing for Familial Hypercholesterolemia (FH) in the United States.

**Consolidated Framework for Implementation Research (CFIR) Domain #1, Characteristics of Intervention**	
Facilitators	Barriers
Well-phenotyped individual (definite or probable diagnosis) with comprehensive family evaluation [10,39]. Pre-test genetic counseling is provided by the ordering physician or genetic counselor [9]. Testing is covered by patient’s third-party or insurance payers (commercial or government insurers) [10]. Simple deoxyribonucleic acid (DNA) sample collection methods (buccal swabs and saliva versus blood) [10]. Overall decreasing cost of genomic sequencing [1,10,39,40]. Financial assistance programs available to patients [10]. Post-test genetic counseling is provided by the ordering physician or genetic counselor [10]. Logistical considerations; short turnaround times to receive results (weeks versus months) [10]. Advances in next-generation sequencing render the discovery of additional novel genes for FH likely [41]. Genetic test results can be interpreted based on established criteria set forth by the American College of Medical Genetics and Genomics [39]. Test results are or can be used to promote or measure changes in patient adherence to lipid lowering therapy [3].	FH diagnosis can be made based on family history and lipid levels despite evidence suggesting the use of FH genetic testing to detect individuals, especially children, who might go undetected using these clinical criteria alone [42]. Clinical diagnostic criteria are not completely fulfilled in index patient [1,10]. Pre-test genetic counseling services are unavailable (due to location or lack of third-party insurance coverage) [10]. FH genetic testing for at-risk relatives is not available when a causal variant is not identified in an index patient [10]. Current costs for testing for common FH-associated pathogenic remain significant ($500–1500) [1]. Testing is not covered by patient third-party or insurance payers (commercial or government insurers) [10]. No financial assistance programs available to patients to cover testing cost(s) [10]. Difficult DNA sample collection method (blood versus buccal swabs and saliva) [10]. Inappropriate utilization of genetic testing- poor phenotyping and inappropriate genetic test selection [39].
**CFIR Domain #2, Outer Setting**	
Facilitators	Barriers
Proximal location of FH screening to allow rapid recruitment [43]. A national expert consensus panel recommended, and current evidence supports the notion, that FH genetic testing become the standard of care for patients with definite or probable FH [9,40]. Collaboration with external centers with specific expertise in cardiovascular genetics [10].	Comprehensive genetic counseling pre-and post-test is unavailable [40]. Providers may have concerns about insurance coverage of FH genetic testing since payers experience barriers to the systematic evaluation of many genetic tests to substantiate coverage [30]. Challenges with index patient identification (proband identification) [1,44]. Cascade screening from an index case; privacy concerns and regulations require that the proband makes the first contact with family members (except in cases of imminent danger) [1,10]. Identification and recruitment of family members [43]. Testing may involve stigmatization or discrimination upon a positive test result (limitations to nondiscrimination protections in the Genetic Information and Nondiscrimination Act) [1,10,40,41].
**CFIR Domain #3, Inner Setting**	
Facilitators	Barriers
Use of electronic health record (EHR) data to facilitate cascade screening and FH genetic testing and counseling in probands [1]. Use of an EHR database that can be queried to identify FH patients with high sensitivity and specificity [45]. Collaboration with internal centers with specific expertise in cardiovascular genetics [10]. Available clinical decision support tools to assist with the evaluation and treatment of FH [46].	Existing diagnostic criteria are not optimized to facilitate FH patient identification via searches of electronic medical records [47]. Clinicians often lack time to completely collect, interpret, and discuss family history of FH during a busy clinical visit [48]. A patient’s self-reported family history is often suboptimal and ranges widely from 30 to 90% accuracy; accuracy depends on the degree of family relatedness and the reported disease [48]. Paper-based and web-based tools to help patients organize their family history outside of the clinical visit are poorly adopted outside of research settings and could be improved [48].
**CFIR Domain #4, Characteristics of Individuals**	
Facilitators	Barriers
Available patient-centric educational tools and decision aids for FH patients and their family members [49]. Use of comprehensive genetic counseling pre-and post- test is available [40]. Established clinical FH diagnosis leads to genetic testing [41]. Individual readiness to make behavioral changes to treat FH [43].	Some providers may perceive genetic testing as out of their scope of practice or area of expertise [50]. Lack of knowledge about FH among patients [49]. Patient concern about having the appropriate communication skills to inform family members about hypothetical FH results [43]. Education gaps or lack of curricula for genetic counselors and other specialists about the genetics and genomics of FH [40,41,47]. Testing may accompany potentially sensitive family and ethical issues [41]. Potential for poor psychosocial functioning due to challenges associated with diagnosis, which affects effective communication of genetic information between proband and his/her family members [10].
**CFIR Domain #5, Process**	
Facilitators	Barriers
International Classification of Diseases, Tenth Revision, Clinical Modification (ICD-10 CM) code for FH diagnosis [45,47]. A centralized genetic screening service with guidelines for insurance reimbursement [41]. Ordering, interpretation, and communication of genetic test results can be done under the guidance of a genetically oriented cardiologist and/or medical geneticist in conjunction with a cardiovascular-oriented genetic counselor [39]. Coordinated efforts exist among general practitioners/general cardiologists, genetic counselors, medical geneticists, and cardiovascular sub-specialists with expertise in FH [39].	Risk-stratification and clinical management decisions in FH are currently and mostly by low-density lipoprotein cholesterol levels and therapeutic response [39].
